# Basic color categories and Mandarin Chinese color terms

**DOI:** 10.1371/journal.pone.0206699

**Published:** 2018-11-28

**Authors:** Vincent C. Sun, Chien-Chung Chen

**Affiliations:** 1 Department of Mass Communication, Chinese Culture University, Taipei, Taiwan; 2 Department of Psychology, National Taiwan University, Taipei, Taiwan; The University of Memphis, UNITED STATES

## Abstract

Basic color terms used in Mandarin Chinese have been controversial since first discussed by Berlin and Kay in 1969. Previous studies showed much inconsistency on what should be considered as basic color terms in Mandarin Chinese. In the present study, we investigated categories of color rather than merely the color terms used by Taiwanese native Mandarin speakers. Using samples conforming to the Berlin and Kay survey, various colors were chosen from a collection of Natural Color System (NCS) colored papers and mounted on a piece of neutral gray card. The card was then mounted on a touch-screen, under D65 illumination. Thirty-two single-character color related Mandarin terms were selected from a Chinese character database according to frequency of use. Participants were required to select the color sample that matched the term by pressing a virtual button on the touch screen. The results show that certain terms can be directly correlated to basic color terms in English, comparable with the results of Berlin and Kay’s original study and those that followed. However, some terms, such as *Mo* (墨 ink), *Tie* (鐵 iron), and *Cai* (菜vegetable), show a wide spread of term maps and inconsistent use among subjects. Principle component analysis (PCA) procedures were used to analysis the commodity of data among subjects. The findings suggest that the basic color categories among Mandarin Chinese speakers are similar to those found in the World Color Survey (WCS), but are represented by wide-spread and inconsistent color terms among speakers.

## Introduction

Modern studies on the basic color terms began with the seminal work of Berlin and Kay [1], who suggested that a basic color term should have four characteristics: (i) its meaning cannot be predicted from its parts; (ii) its meaning is not included in that of another term; (iii) it is not specific to a narrow class of objects; and (iv) it must be psychologically salient for the users. Berlin and Kay applied these criteria to determine the basic color terms in several languages [1, 2]. They reported that in the English language, there were eleven basic color terms: white, gray, black, blue, green, red, yellow, orange, brown, purple and pink. In the same study, Berlin and Kay [1] reported that Mandarin Chinese had six basic color terms.

However, their conclusions regarding Mandarin Chinese were controversial from the very beginning. In an earlier version of their report [3], gray was listed as a basic color term in Mandarin. However, according to their evolution theory of color naming, this listing was an anomaly for the theory proposes that as a civilization evolves, the basic color terms increase in a specific chronological order and gray is one of the last terms to emerge. Therefore, it is difficult to explain why it appears as a basic color term while the terms that emerge in an earlier stage, such as brown, do not. Berlin and Kay [1] resolved this problem by removing a Taiwanese respondent from the data set as other Mandarin respondents treated gray as a secondary color term.

Lü [4], who surveyed 1,815 local respondents in Taiwan and collected more than 1600 color terms, reported that there were terms for each of the eleven basic color terms proposed by Berlin and Kay [1]. Lin et al. [5, 6], applying a similar unconstrained method as Berlin and Kay [1] and Lü [4], suggested that there were eleven basic color names in Mandarin Chinese. These were used in the same way as the corresponding terms in British English and therefore matched the eleven found by Berlin and Kay [1]. However, Gao and Sutrop [7], using a similar approach, showed that there were nine basic color terms in Mandarin Chinese with orange and brown missing from Berlin and Kay’s [1] eleven. Using a more quantitative approach, Uchikawa [8] found that of the eleven basic color terms, those used most consistently are gray, blue, green, red, yellow, purple, and pink; less consistently used are brown, orange, white and black.

The discrepancies in what comprises the basic Mandarin color terms may be resulted from a particular characteristics of Mandarin Chinese in that there is more than one synonym for each color. Different respondents may just use different terms for the same color. This variety of color terms makes it difficult to determine the basic color terms according to the criteria suggested by Berlin and Kay [1]. For instance, Uchikawa [8] and Gao and Sutrop [7] identified that while there were two terms for brown in Mandarin (*Zong* and *He*), each respondent may consistently use just one or the other to name the brownish chips. Hence, in the total population there was little consistency in the naming of brownish chips even though each of the two terms may be salient for an individual. As a result, brown was excluded from basic color terms for failing Berlin & Kay’s [1] criteria of saliency. Later researchers who were either native Mandarin speakers themselves or had native Mandarin speakers as colleagues corrected their data, based on their first-hand understanding of the language [4, 5, 7], suggested more basic color terms than those of Berlin and Kay [1]. The discrepancies among those research findings could be caused by multiple color terms used in Mandarin for a single category of color. However, although factoring in multiple color terms for the same perceived colors may be a linguistically sound process, it still requires empirical justification. Thus the purpose of this study is to test whether these terms refer to the same color categories.

Many studies have described the basic color terms according to color categories [9–15] without distinguishing between the two. These studies showed that basic color terms could be just labels for basic color categories, which were common cognitive structures among users of a language. In the studies of Mandarin color terms, Lin et al. [6] used the basic color terms as indexes to categorize color samples. Thus, while Mandarin speakers may show inconsistency in the use of color terms, their reference to color categories may be consistent. However, assessing this is not possible by the conventional method for color terms, which is to collect the terms used for various color samples. For instance, one respondent may use only *Zong* for brown while the other, *He*. The researcher would not know whether each was using a different word for the same color category or two overlapped color categories with the test sample as the intersection. In the current study, we used a different approach. Instead of asking participants to give color terms for a color sample, we gave them color terms and measured what color samples were associated with each term. If two color terms were synonyms for the same color category, the participants should choose similar sets of color samples as representations for these terms. Thus, the participants’ response to these terms would be highly correlated. Such correlation should be able to be detected by a factor analysis. Thus, our paradigm should be more suitable for establishing the association between color samples and color terms when individuals use a variety of words.

## Methods

### Participants

Sixty-three participants, with ages ranging from 20 to 30 years, participated in this experiment. The participants were recruited from the National Taiwan University (NTU) and the Chinese Culture University (CCU), with the experiment being conducted on each of the two campuses. All participants were native Mandarin speakers and with normal color vision as examined with an Ishihara Test. All of them were with normal or corrected to normal vision acuity during the survey. The use of human participants was approved by the Research Ethics Committee of National Taiwan University. Written consent to participate in this study was obtained from each participant. A participant could refuse to sign the form and leave with no consequences.

### Color samples

**Fig 1:**
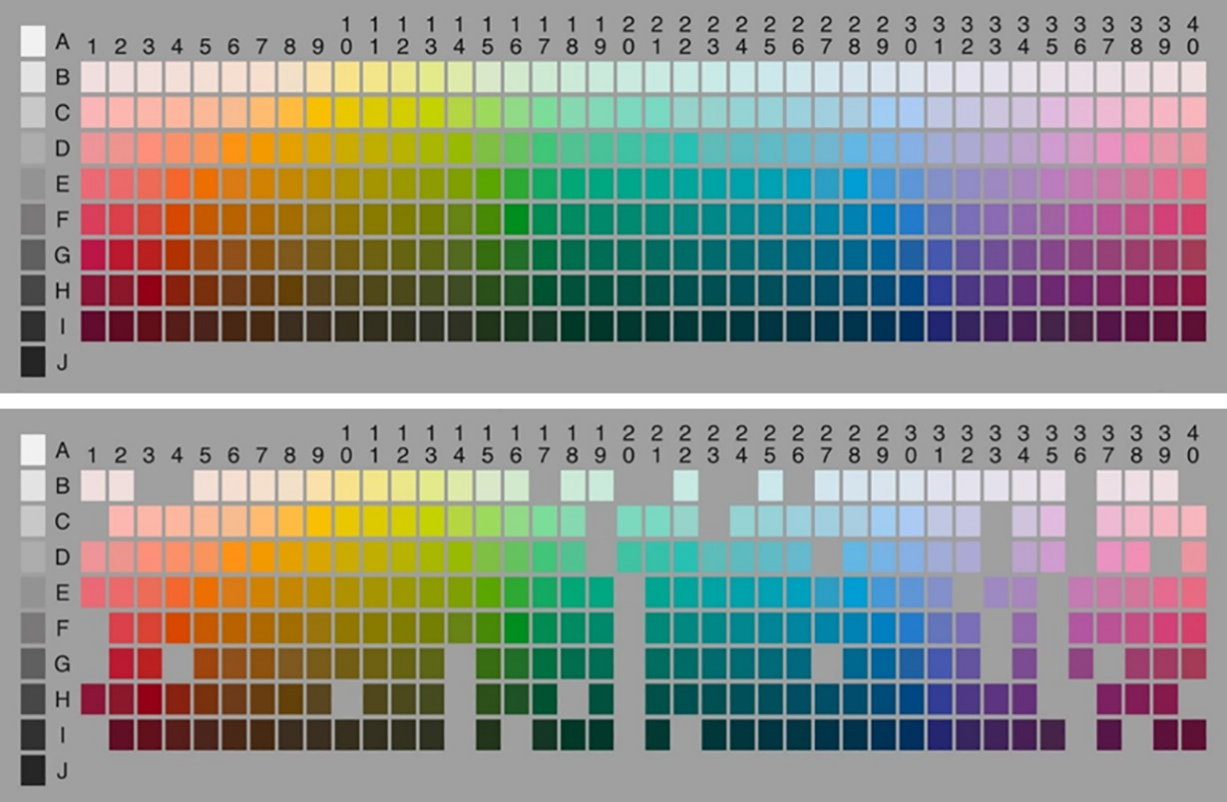
The 280 color samples used in the current study (upper panel) when listed according to the color sample palette used in the WCS (lower panel). The color samples were displayed on three cards for the survey (shown in Fig 2).

**Fig 2:**
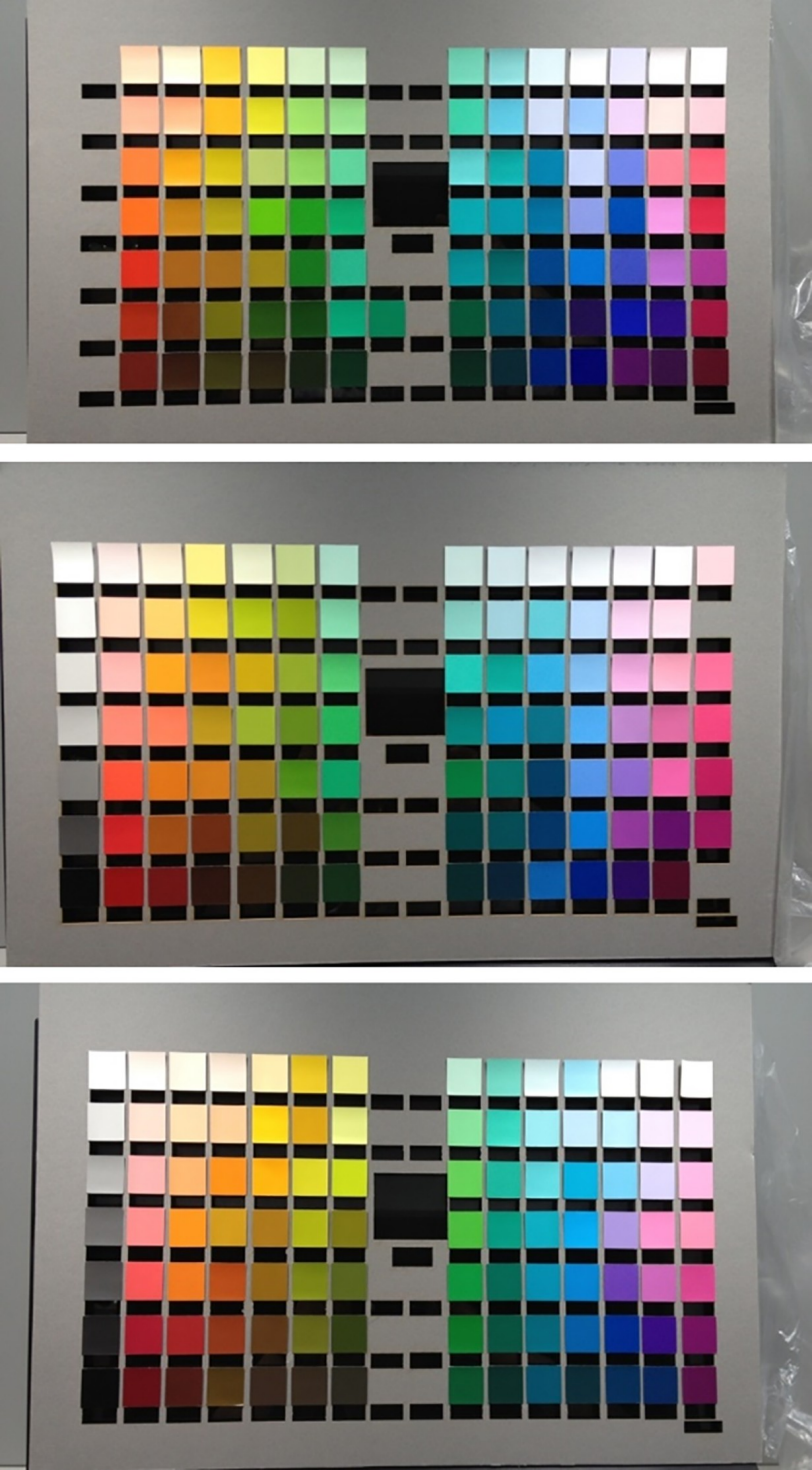
The color samples presented during the survey: color chips mounted on cards.

In total, 280 color samples were used as the reference samples for the experiment. These were 25.4 mm squares of paper from the Natural Color System (NCS) and were selected to match the color samples used in the World Color Survey [2] (Fig 1). The samples were mounted on neutral gray cards, 0.55 m (width) by 0.45 m (height) in dimension. The cardboards were cut to fit the size of a touch screen. The cardboards had a 55 mm by 50 mm rectangular opening at the center, and the samples were evenly distributed around the cardboards. There was a slit below each color sample, as shown in Fig 2; it allowed the participants to make a response on the touch screen. Since each cardboard could hold only 106 color samples, the samples were separated into three groups with each group being mounted on one cardboard. Samples were matched for lightness and chromatics for each cardboard according to the World Color Survey [2] (WCS) stimulus palette.

### Mandarin color terms

For the test, thirty-two single-character Mandarin words were selected from 4430 of the most frequently used characters in the Academia Sinica Balanced Corpus [16] (Table 1). We used only single-character words to ensure the test material met the monolexical criterion of Berlin and Kay [1] and we used only medium- to high-frequency words to fulfill the saliency criterion. Eleven of these were Lü’s [4] Mandarin basic color terms. Some words did not appear on the list of basic color terms but were considered to be synonyms of the eleven terms. For example, *Chi* (赤), *Zhu* (朱), and *Dan* (丹) all mean RED in Mandarin, and *Zong* (棕) and *Zhe* (褚) both mean BROWN. Some terms are names of common objects but are also commonly used to describe the color of another object. An example is *Cha* (茶), or ‘tea’, which is often used by Mandarin speakers to describe a brownish color. The rest are modifiers that can be associated with many colors and often are not used as an independent term. One example is the word *Dan`*(淡) or ‘pale’ which can be linked to many other color terms to form a compound word for the unsaturated version of that color. With basic color terms, we expected that the participants would have a highly concentrated selection in a handful of samples. However, if the number of selected samples is limited, it would be difficult to discern whether it is due to a lack of motivation for the participants to pick up more samples or the specialization of the color category. On the other hand, the modifiers, which can be associate with many colors, should allow the participants to select a wide range of color samples and thus can be used a diagnostic tool for the former issue. In indeed, as reported in Result, the participants did make a widespread choice of samples for the modifiers and concentrated ones for the basic color terms. Thus, it is an evidence that our participants made their best effort for the task.

**Table 1:** Mandarin Chinese words used in the study, in Pinyin alphabetical order.

#	Chinese Pinyin System	Classical Chinese Font	Direct English Translation (& associated color)
1	Bai	白*	White
2	Bi	碧	Jade (Green)
3	Cai	菜	Vegetal (Green)
4	Cha	茶	Tea (Brown)
5	Cheng	橙*	Orange
6	Chi	赤	Red
7	Cui	翠	Fresh Green
8	Dan	丹	Red
9	Dan`	淡	Pale
10	He	褐*	Brown
11	Hei	黑*	Black
12	Huang	黃*	Yellow
13	Hong	紅*	Red
14	Hui	灰*	Gray
15	Jin	金	Gold (Golden)
16	Ju	菊	Daisy (Yellow)
17	Lan	藍*	Blue
18	Lu	綠*	Green
19	Mo	墨	Ink (Black)
20	Qing	青	Blue-Green
21	Shui	水	Water (Light Blue)
22	Tao	桃*	Peach (Pink)
23	Tie	鐵	Iron (Gray)
24	Tu	土	Earth (Brown)
25	Xie	血	Blood (Red)
26	Xuan	玄	Weird (Black)
27	Yin	銀	Silver (Shiny Gray)
28	Ying	櫻	Cherry (Pink)
29	Zhe	褚	Brown
30	Zhu	朱	Red
31	Zi	紫*	Purple
32	Zong	棕	Brown

*The Mandarin basic color terms suggested by Lü (1997).

### Procedures

At the beginning of the survey, each participant’s color vision was tested using an Ishihara pseudoisochromatic test to make sure that no participant had defective color vision. All participants were found to have normal or corrected-to-normal visual acuity.

**Fig 3:**
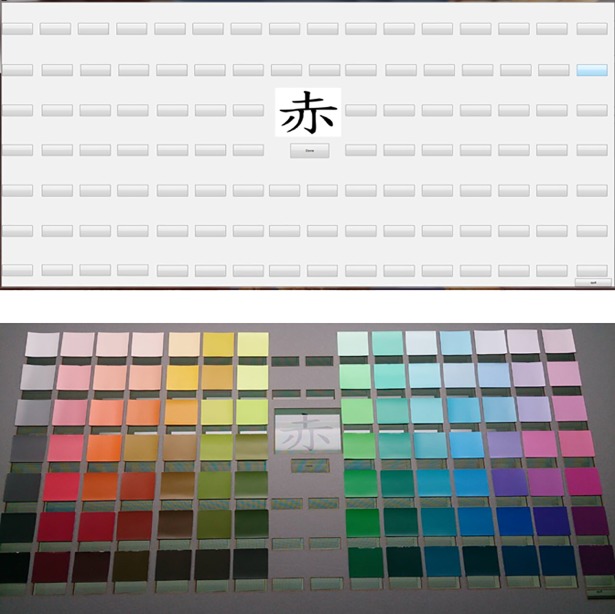
Screen shot of the touch screen monitor. Cards on which color chips had been mounted were attached to it for the survey, as shown.

The experiment was conducted in either a darkroom (NTU) or in a room with reduced lighting (CCU). The former was originally designed for dark adaptation experiment and thus was practically light-proof while the latter, though with lights turned off and the door closed, still had residual illumination leaked through the silt at the bottom of the door from the adjacent office. In the room, the cardboards with their color samples were displayed on a 24-inch touch-screen monitor. The monitor was controlled by using a Matlab program on a personal computer. Words were presented in the opening at the center of the cardboard and simulated buttons were displayed on slits below the color chips (Fig 3). The color samples in both test sites were illuminated by standard D65 light sources (Macbeth Spectrolite).

During the experiment, one test word was displayed at a time in a random order. The participants were instructed to pick the color that best corresponded to the test word by pressing the virtual buttons. The selections were automatically recorded. After the color had been selected and recorded, the next word was presented and the procedure repeated. After the survey for the samples on one cardboard had been completed, a different cardboard was presented and the procedure repeated. Due to a programming glitch, the word *Zong* was displayed twice. The results of the two presentations were averaged.

## Results

Of the 63 participants who agreed to take part in the experiment, four did not finish the survey for various reasons. Data from the remaining 59 participants are reported here. For data analysis, we first pooled the responses from all participants together and counted the total number of times, or frequency, a color sample was selected for each color term. The acquired frequency matrix was analyzed in two ways. First, we mapped the frequency matrix to WCS palette. The frequencies for WCS colors not sampled in this study (see Fig 1) were filled with a bilinear interpolation from neighboring data. The result is the frequency distribution presented below. Second, we perform a principle component-based factor analysis the original frequency matrix. The rationale is that if two terms referred to the same color category, the participants’ response to them would be highly correlated. Thus analyzing the correlation structure of the frequency matrix should help us to identify the synonyms. The factor loadings of tested color terms on major factors are reported below.

**Fig 4:**
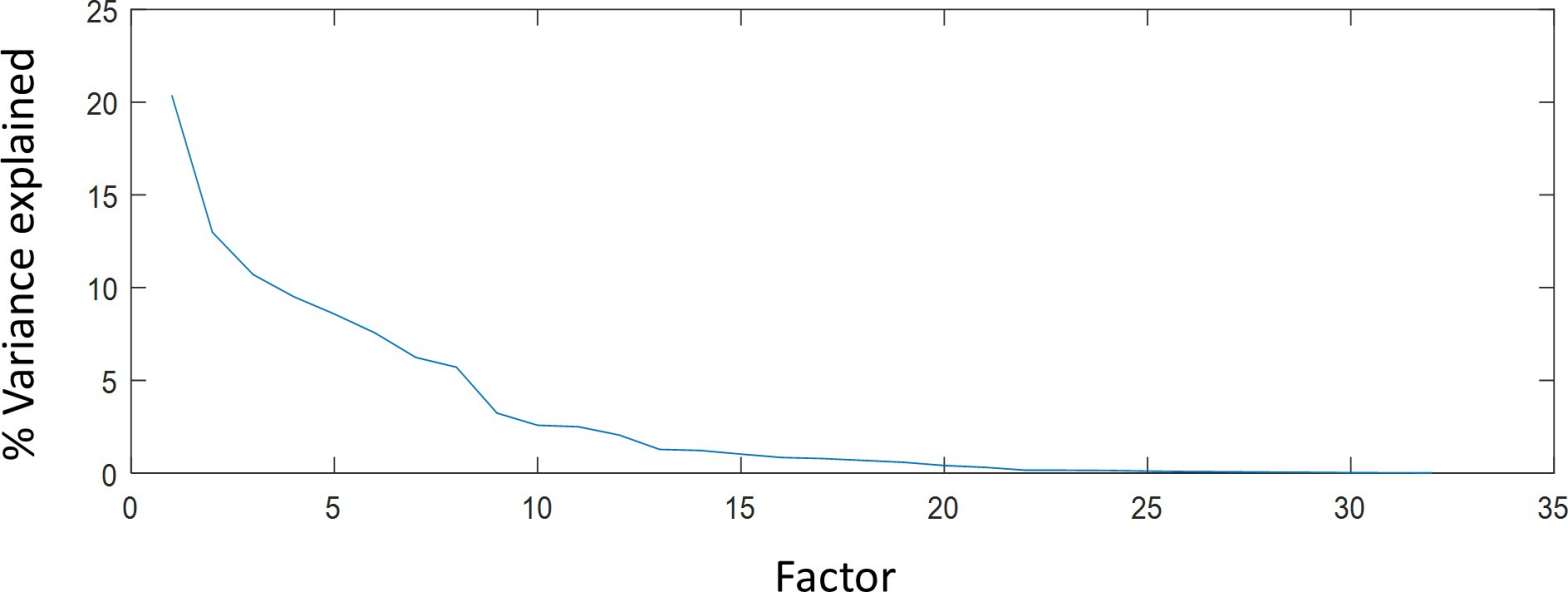
The proportional variation in the summed data of all subjects that can be explained by each factor, sorted by the amount of variance explained.

**Fig 5:**
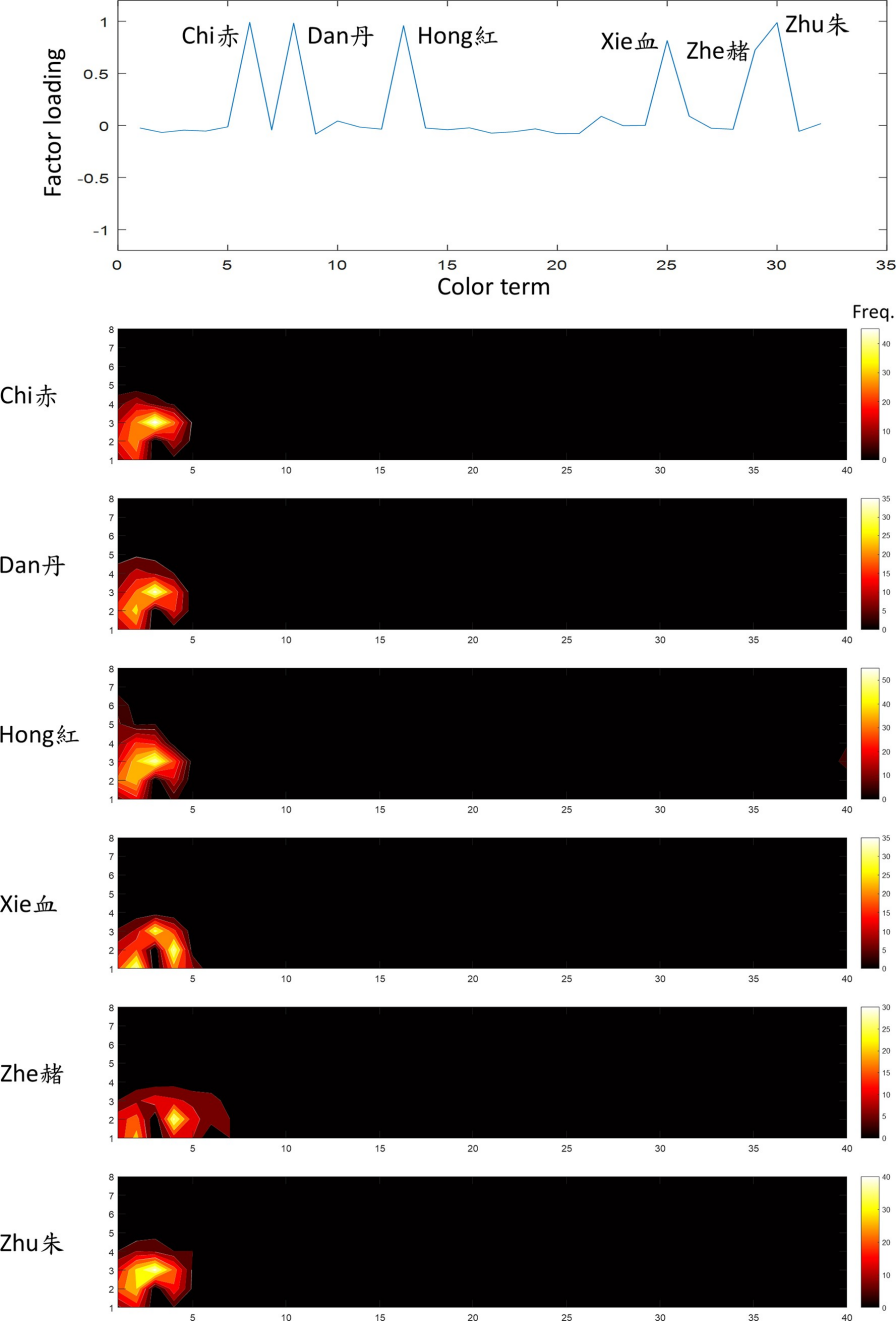
The first factor was “red”. The upper most panel shows the factor loading (ordinate) of each color name (abscissa). The number on the abscissa, from 1 to 32, shows the sequence of the test words as listed in Table 1. The terms *Chi*, *Hong*, *Zhu*, *Zhe*, *Dan* and *Xie* (赤,紅,朱,赭,丹,血) had high loading on this factor. The lower panels each shows the frequency distribution on WCS charts of one of the highly loaded term. The numbers 1 to 8 on the left of WCS charts are lightness value, equivalent to the B through I indexes of a typical WCS chart [17], and the numbers at the bottom (1 to 40) indicate hues, as shown in Fig 1. The color scale on the right of each chart represents the summed frequency of sample selections.

**Fig 6:**
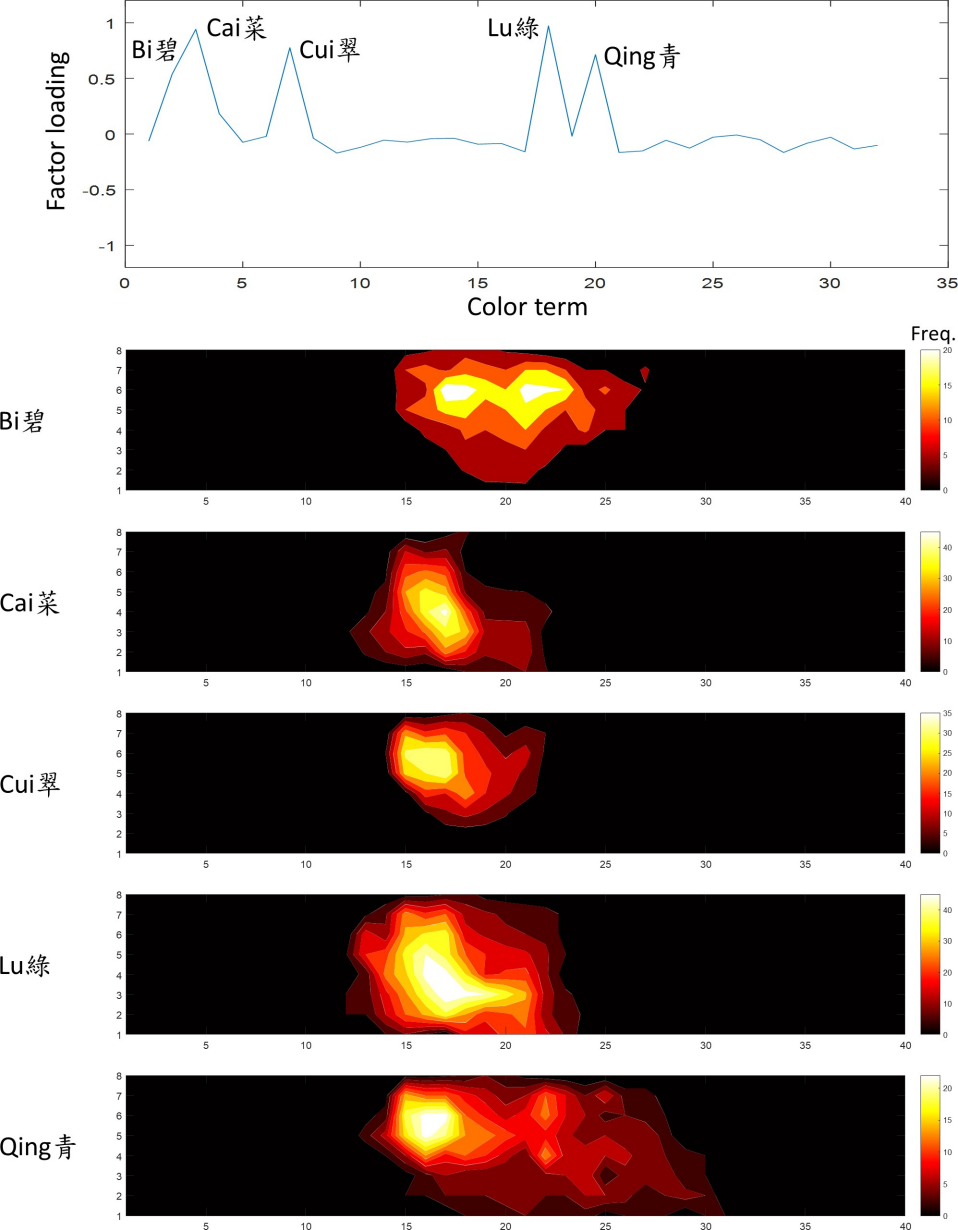
The second factor was “brown”. The terms *He*, *Zong* and *Tu* (褐,棕,土) showed had high loading on this factor. The convention of the figure is the same as Fig 5.

**Fig 7:**
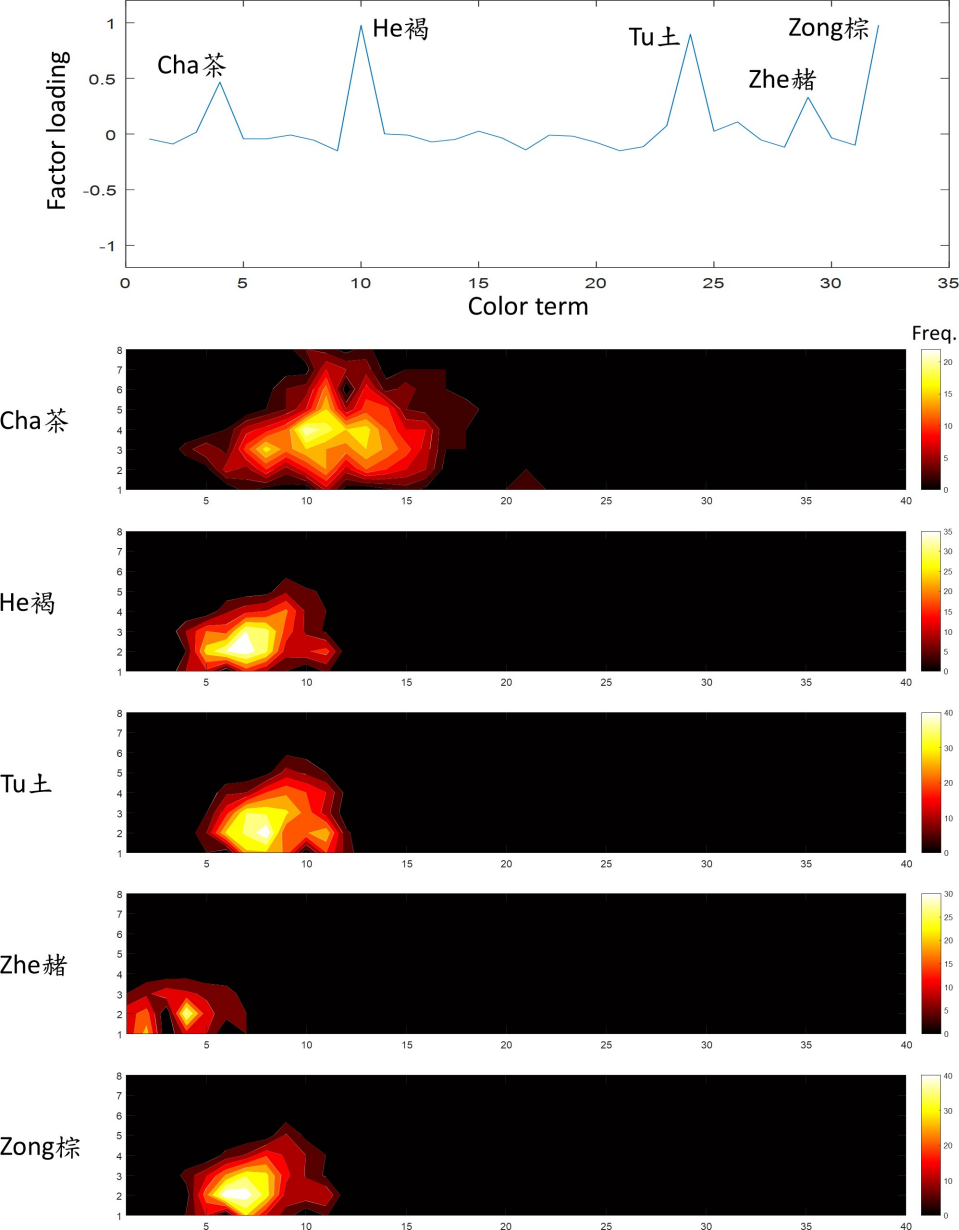
The third factor was “green”. The terms *Cai*, *Cui*, *Lu*, *Bi* and *Qing* (菜,翠,綠,碧,青) showed a high loading on this factor. The convention of the figure is the same as Fig 5.

**Fig 8:**
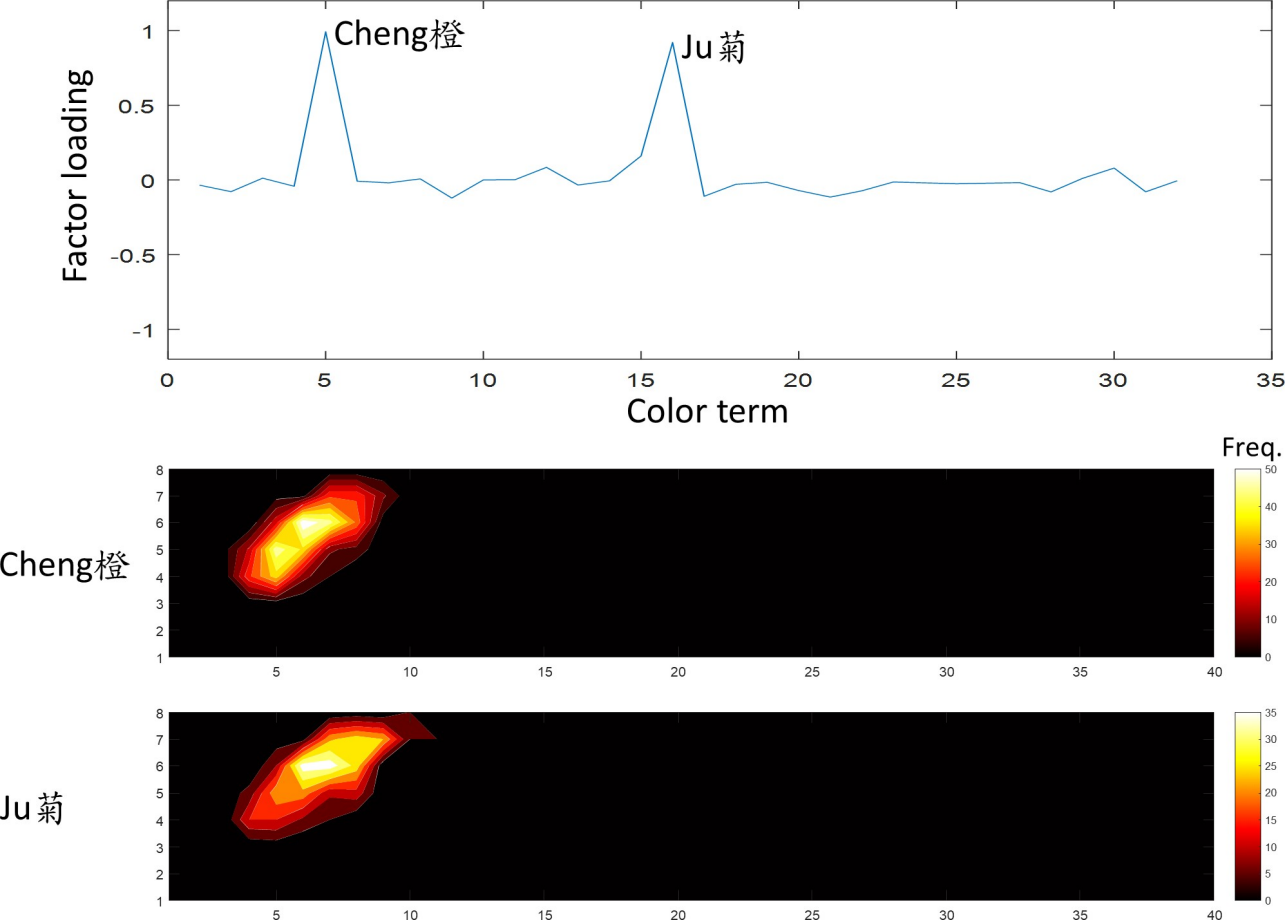
The fourth factor was “black”. The terms *Hei*, *Mo* and *Xuan* (黑,墨,玄) showed a high loading on this factor. The convention of the figure is the same as Fig 5.

**Fig 9:**
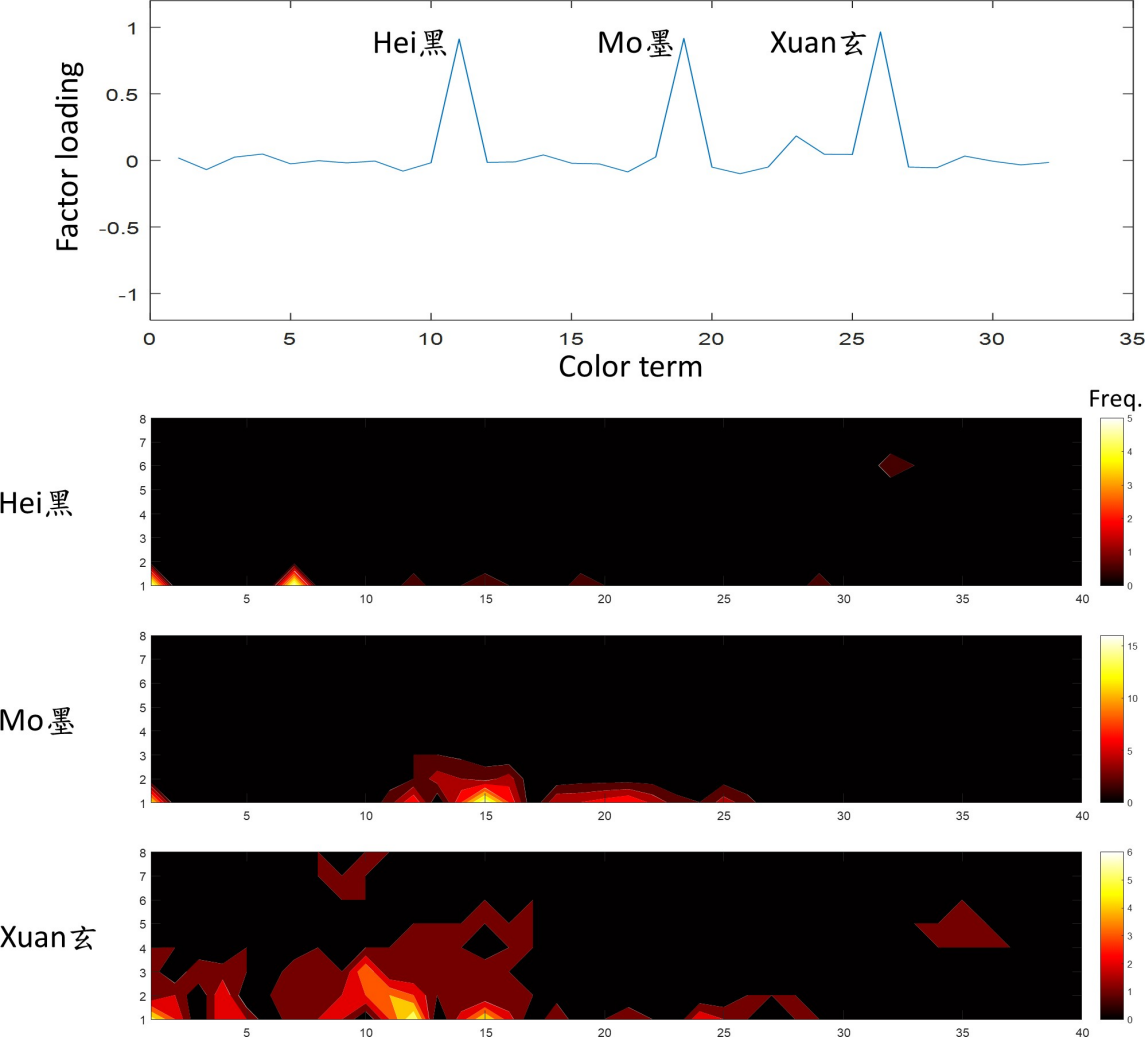
The fifth factor was “orange”. The terms *Cheng* and *Ju* (橙,菊) showed a high loading on this factor. The convention of the figure is the same as Fig 5.

**Fig 10:**
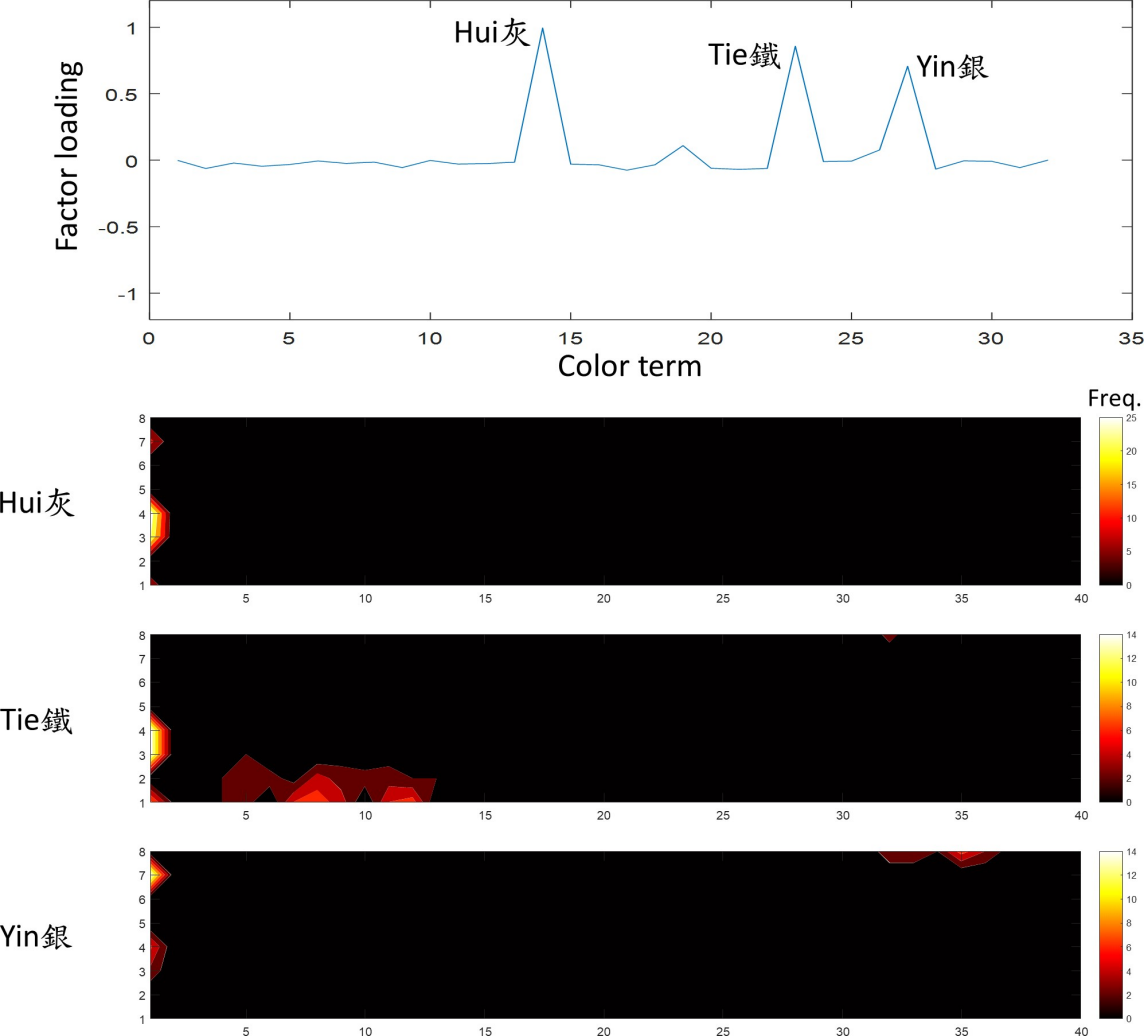
The six factor was “gray”. The terms *Yin*, *Te*, and *Hui* (銀,鐵,灰) showed a high loading on this factor. The convention of the figure is the same as Fig 5.

**Fig 11:**
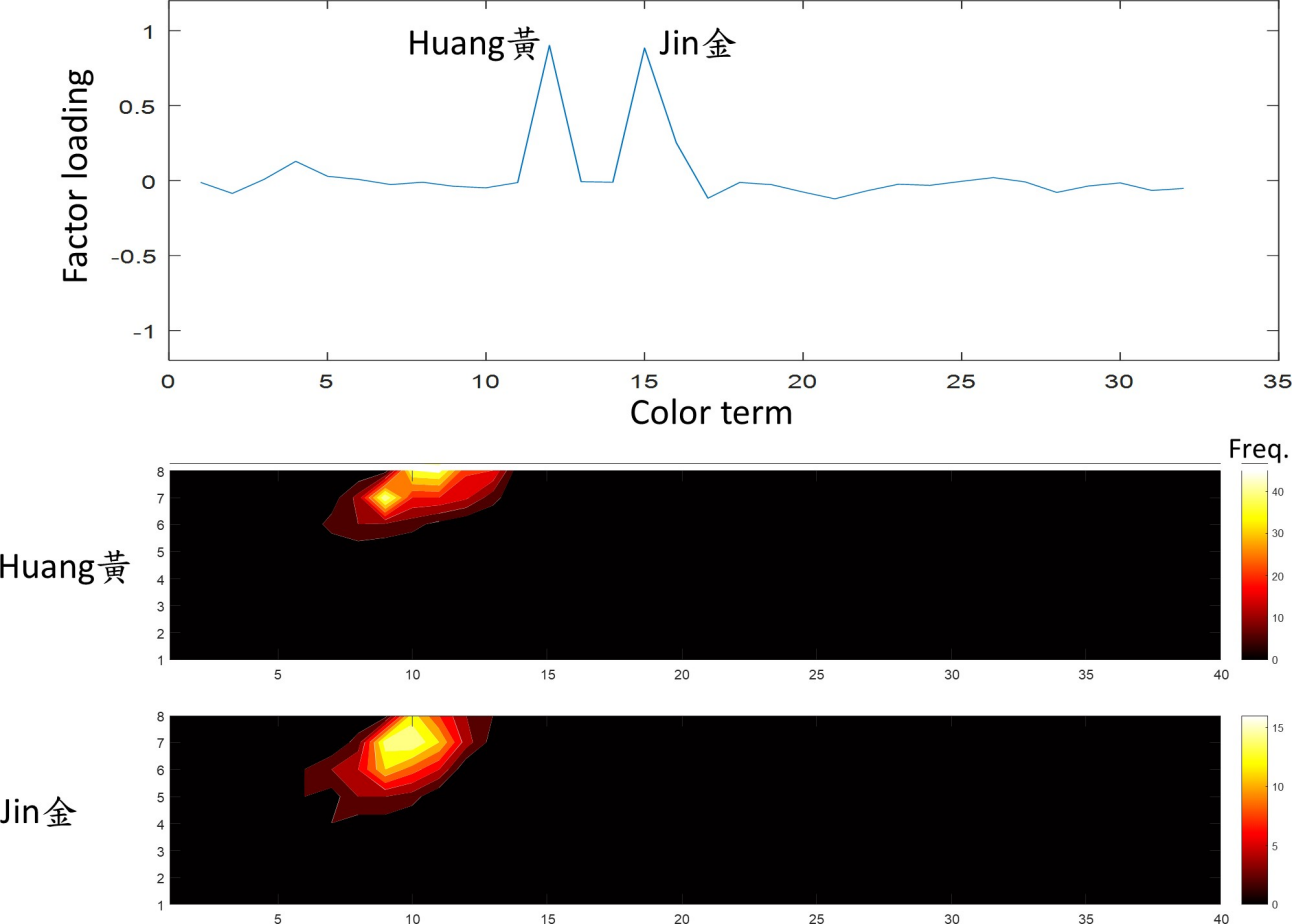
The seventh factor was “yellow”. The terms *Huang* and *Jin* (黃,金) showed a high loading on this factor. The convention of the figure is the same as Fig 5.

**Fig 12:**
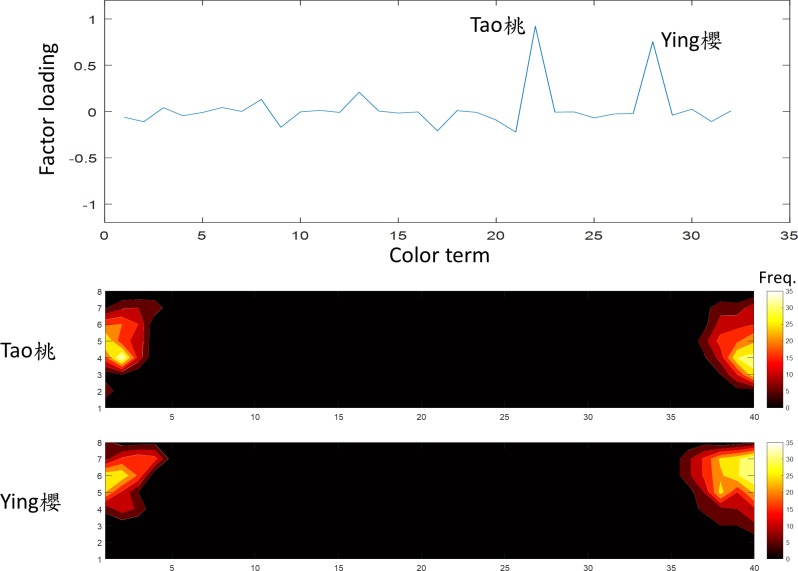
The eighth factor was “pink”. The terms *Ying* and *Tao* (櫻,桃) showed a high loading on this factor. The convention of the figure is the same as Fig 5.

Fig 4 shows that the proportional variation in the data can be explained by each factor, computed as the Eigen value corresponding to that factor divided by the sum of all Eigenvalues, sorted by the amount of variance. It is obvious that there is an “elbow” between components #8 and #9. The factors beyond #8 explain little about the correlation structure in the data. Based on this, we first investigated the first eight factors. Figs 5–12 summarize the loading of each term in those factors and the frequency distribution of the highly loaded terms on a WCS stimulus chart.

The first factor, explained 21.2% of variation in the data, was the “red” family. The terms *Chi*, *Hong*, *Zhu*, *Zhe*, *Dan* and *Xie* (赤,紅,朱,赭,丹,血) all had high loading on this factor (Fig 5). The term *Hong* is a typical translation of “red” in Mandarin. The frequency distributions for *Chi*, *Zhu*, *Dan* and *Xie*, are virtually almost identical to *Hong*. This provides evidence that *Hong*, *Chi*, *Zhu* and *Dan* are indeed synonyms for the color of blood (*Xie*). The frequency distributions for the secondary term *Zhe* extended to the brown region, as one would expect from its meaning of reddish brown.

The second factor, explained 16.9% of variation in the data, was the “brown” family. The terms *He*, *Zong* and *Tu* (褐,棕,土) showed a strong loading on this factor (Fig 6). These three terms had a very similar frequency distribution to “brown” in English on the WCS chart, suggesting that they are all synonyms for the color of mud (*Tu*) or the bark of a pine tree (*Zong*). The secondary terms *Zhe* (赭 reddish brown) and *Cha* (茶 tea, thus brownish green) also showed loading on this factor. Their frequency distribution covered extensive regions overlapping the “brown” region but also extended to “red” or “green” for *Zhe* and *Cha* respectively.

The third factor, explained 11.8% of variation in the data, was the “green” family. The terms *Cai*, *Cui*, *Lu*, *Bi* and *Qing* (菜,翠,綠,碧,青) were heavily loaded on this factor (Fig 7). *Lu* is a typical Mandarin translation of “green”. The frequency distribution of *Cai*, *Cui*, and *Lu* were very similar, suggesting that they are synonyms for the color of the vegetable (*Cai*). The frequency distribution for the secondary terms *Bi* (Jade) and *Qing* extended to the blue region, suggesting “blue-green”.

The fourth factor, explained 9.9% of variation in the data, was the “black” family. The terms *Hei*, *Mo* and *Xuan* (黑,墨,玄) were heavily weighted on this factor (Fig 8). *Hei* is a typical translation of “black”. Virtually all participants selected the darkest monochromatic sample when prompted by *Hei* and *Xuan*. In addition to black, *Mo* can be associated with another color term for the dark version of that color. Thus, it is not surprising that its frequency distribution covered a wide range of dark color samples.

The fifth factor, explained 8.1% of variation in the data, was the “orange” family. The terms *Cheng* and *Ju* (橙,菊) showed high loading on this factor and had almost identical frequency distributions to the WCS chart (Fig 9). Thus, these two terms are indeed synonyms for the color of an orange (*Cheng*).

The sixth factor, explained 6.1% of variation in the data, was the “gray” family. The terms *Yin*, *Te*, and *Hui* (銀,鐵,灰) all had a large loading of this factor (Fig 10). Their frequency distribution covered the intermediate achromatic region. *Hui*, a typical Mandarin translation of gray, covered a wide range of the achromatic region. *Yin* (silver) covered mostly the bright achromatic region while *Te* (iron) covered mostly the dark achromatic region. However, since *Te* can also be used as a modulator, it is not surprising that it was identified with a wide range of dark colors.

The seventh factor, explained 4.7% of variation in the data, was the “yellow” family. The terms *Huang* and *Jin* (黃,金) were heavily weighted on this factor and had a similar frequency distribution (Fig 11), suggesting indeed that *Huang* refers to the color of gold (*Jin*).

The terms *Ying* and *Tao* (櫻,桃) were heavily weighted on the eighth factor, which explained 4.4% of variation in the data,. Their frequency distributions were almost identical (Fig 12) and occupied the same regions as “pink” in English (1). Thus, this factor represents pink.

Twenty-seven of the thirty-two color terms showed high loading in one of the eight factors above. Of the five color terms not among these eight factors, the terms *Lan* and *Shui* (藍,水) showed up in the tenth factor. The frequency distribution of *Lan* occupied the same region as “blue” in English. *Zi* (紫) was in the twelfth factor and occupied a region similar to English “purple” and did not overlap any other color terms in our stimulus set. *Bei* (白), in factor 13, was for the lightest achromatic sample. *Dan*、(淡), however, occupied a wide range at the top of the WCS chart, consistent with its use as a modulator for the unsaturated version of any color.

It is important to note that exclusion from one of the first factors does not imply a color term is not important. The first factors are those that can explain the largest variability in the correlational structure of the color terms. As shown above, factor analysis is useful for identifying synonyms or overlapping terms. This suits one of the purposes of this study, which was to show that there are many synonyms for color terms. However, a color term that has no synonyms or does not overlap any other terms will constitute a factor that explains very little variability in the correlation structure. Thus, it would be excluded from the first factors even though it may an important basic term, as the case of Bei, a typical translation of white, and Lan, a typical translation of blue.

## Discussion

**Fig 13:**
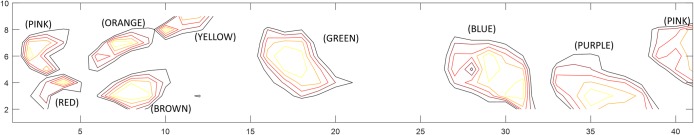
The pooled frequency distribution of the synonyms for color categories on the WCS chart.

In this study, we used a paradigm that is very different from the conventional methods used since Berlin and Kay’s research. That is, rather than showing color chips to an informant and inquiring about the corresponding color terms, we showed participants a color term and asked them to identify all the color samples in our stimulus set that matched the term. We hypothesized that much of the controversy about the basic color terms in Mandarin Chinese had to do with the large number of synonyms for each color category. As a result, previous surveys seemed to offer little consistency among respondents. We found there are many synonymous terms for color categories such as red, green, blue, yellow, brown, pink, orange, black, and gray, as listed in Table 2. Because these synonyms showed virtually an identical distribution on the WCS chart, we merged the frequency distribution of the synonyms to plot the boundaries of color categories on the WCS chart (Fig 13).

**Table 2:** Basic color categories, denoted by basic color terms, and their typical terms and synonyms in Mandarin Chinese. The number in each parentheses is the sequence number of each word in Table 1.

Basic color term	Typical Mandarin term	Synonyms
Black	Hei (10)	Xuan (26)
White	Bai (1)	
Red	Hong (13)	Chi (6), Zhu (30), Dan (8), Xie (25)
Green	Lu (18)	Cai (3), Cui (7)
Blue	Lan (17)	
Yellow	Huang (12)	Jin (15)
Brown	He(10), Zong (32)	Tu (24)
Orange	Cheng (5)	Ju (16)
Pink		Ying (28), Tao (22)
Purple	Zi (31)	
Gray	Hui (14)	

In some categories, there was one dominant term that is most frequently used. For instance, for “red”, the frequently used *Hung* ranked 438 among 4430 of the most commonly used characters according to Academia Sinica Balanced Corpus [16], while the alternatives, *Chu* and *Chi*, ranked 1304 and 1527 respectively. When such a dominant term exists, there would be little inconsistency among the respondents, as everyone would use the same term for that color category. The color categories for green, yellow, blue, black, white and orange also have one dominant term.

However, participants’ responses will be very inconsistent when two terms are used with almost the same frequency. One such example is “brown”. Berlin and Kay [1] suggested that brown is not a basic color term in Mandarin Chinese. We found that three terms, *Zhe*, *Zong*, and *Tu* occupied the same region on the WCS chart as brown in English and that this region does not overlap other basic color terms. Thus, it should also be a basic color term. The issue here is that *Zhe* and *Zong*, with almost the same frequency of use, rank 2468 and 2598 respectively among native Mandarin speakers according to Academia Sinica Balanced Corpus [16]. *Tu’*s much higher frequency of use is due to its meaning “mud” or “earth” rather than color. Individuals seem to have their preference. As a result, as suggested by Uchikawa [8], there is little consistency when the results of all respondents are combined.

“Gray” was another controversial basic color term in Berlin and Kay’ [1]. They even removed a respondent who used gray as a basic color term from their data set to avoid an anomaly in their data. We found that there are terms (*Hui*, *Yin*) for the achromatic samples in the intermediate brightness range that do not overlap with black (*He*) or white (*Bai*). Thus, gray should be considered as a basic color category in Mandarin Chinese.

Another controversial term is “pink”. The common translation of pink in Mandarin Chinese is *Feng Hung* (粉紅), which is a compound word from *Feng* (powdered) and *Hung* (red). Since the term *Feng* can be associated with other color terms, such and *Lan* (blue) or *Lu* (green), for an unsaturated version of that color, such as *Feng-Lan* or *Feng-Lu* respectively, it may be that Mandarin Chinese speakers just treat pink as a kind of unsaturated red. Thus, it is not considered to be a basic color term by Berlin and Kay [1]. Here, we found two terms (*Ying* and *Tao*) occupied the same region on the WCS chart as pink in English and this region was not covered by any other color terms. Thus, it should represent an independent color category for Mandarin speakers. Perhaps, the term *Feng Hung*, as a common translation of pink, is no longer just unsaturated red in modern usage but refers to a unique color category represented by *Ying* and *Tao* in our data and thus should be considered to be a basic color term.

In sum, we identified eight major factors for color categories: red, brown, green, black, orange, gray, yellow, and pink. The color samples associating with these factors occupy the same regions in the WCS chart as the corresponding basic color terms in English. Thus, these categories, even those with controversies in the literature, such as brown and gray and pink, should have no problem to be considered as basic color categories. The rest three of the eleven basic color terms of Berlin & Kay [1], white, blue and purple did not explain enough variance in our data to be qualified as major factors. However, it is mostly likely due to a lack of synonyms of those terms in our stimulus set than a non-basic term status. The evidence is that the color samples for each of those terms occupies a region that does not overlap with any other basic color terms and is similar to the region of the corresponding color category in English (Fig 13). Furthermore, there is never a controversy in the literature on the status of white and blue as basic color terms in Mandarin. Thus, our data supports the notion that the basic color categories in Mandarin are similar to that in English, as shown in Fig 13.

## Conclusion

Biggam [18] suggested that there is a crucial difference between basic color terms and basic color categories. A color category as a concept belongs to the cognitive domain and color terms and words belong to the linguistic domain. Though the disparity between categories and terms was suggested, the phenomenon of multiple synonyms for a single category in a specific language was not discussed. The present study shows that this phenomenon is typical in Mandarin Chinese. Multiple color synonyms clouded previous searches for basic color terms but did not do so for basic color categories with the factor analysis methodology we used in the present study. These basic categories that can be translated into basic color terms in English have concentrated response maps comparable to the result of WCS.

For terms that are not related to a certain basic category, the data show wide spreads of response on the WCS chart and are inconsistent among subjects.

When identifying basic color categories, it is not necessary to first find the exact basic color terms before finding their corresponding color samples. The findings of the current study suggest that arbitrary words, whether or not they are related to color, or even symbols, pictures and images, can be used as referents for surveying corresponding color samples. Using the standard principle component analysis (PCA) procedure, data analysis can reveal the basic color categories. However, a complete palette of color samples is important for effective data collection. To reduce confusion in the selection of the color sample to a minimum, the color palette should include a complete set of surface colors with uniform perceptual distribution. The methodology could also be used for surveying cognitive categories other than color.

## Supporting information

S1 File The frequency distribution of each color term. Each sheet of the attached file shows the frequency distribution on WCS samples for one color term listed in Table 1. The rows denote lightness while the columns, hue, as shown in Fig 1. The first, or the achromatic, column has ten lightness level while the other columns have eight lightness levels in Row 2–8. Each cell shows the number of the respondents picking the corresponding chip for the designated color term.
(XLS)
